# Ribonucleotide reductase inhibition restores platinum-sensitivity in platinum-resistant ovarian cancer: a Gynecologic Oncology Group Study

**DOI:** 10.1186/1479-5876-10-79

**Published:** 2012-04-27

**Authors:** Charles Kunos, Tomas Radivoyevitch, Fadi W Abdul-Karim, James Fanning, Ovadia Abulafia, Albert J Bonebrake, Lydia Usha

**Affiliations:** 1 Department of Radiation Oncology, University Hospitals of Cleveland, Cleveland, OH 44106, USA; 2 Department of Epidemiology and Biostatistics, Case Western Reserve University, Cleveland, OH 44106, USA; 3 Department of Pathology, University Hospitals of Cleveland, Cleveland, OH 44106, USA; 4 Department of Obstetrics and Gynecology, Division of Gynecologic Oncology, Hershey Medical Center, Hershey, PA 17033, USA; 5 Department of Obstetrics and Gynecology, Division of Gynecologic Oncology, SUNY Health Science Center, Brooklyn, NY 11203, USA; 6 Department of Obstetrics and Gynecology, Division of Gynecologic Oncology, Cancer Research for the Ozarks-Cox Health, Springfield, MO 65807, USA; 7 Department of Medicine, Division of Hematology and Oncology, Rush University, Chicago, IL 60612, USA; 8 Department of Radiation Oncology, University Hospitals of Cleveland, 11100 Euclid Avenue, LTR 6068, Cleveland, OH 44106, USA

**Keywords:** Ovarian cancer, Ribonucleotide reductase, Triapine, Methemoglobinemia

## Abstract

**Background:**

The potent ribonucleotide reductase (RNR) inhibitor 3-aminopyridine-2-carboxyaldehyde-thiosemicarbazone (3-AP) was tested as a chemosensitizer for restored cisplatin-mediated cytotoxicity in platinum-resistant ovarian cancer.

**Methods:**

Preclinical in vitro platinum-resistant ovarian cancer cell survival, RNR activity, and DNA damage assays were done after cisplatin or cisplatin plus 3-AP treatments. Six women with platinum-resistant ovarian cancer underwent four-day 3-AP (96 mg/m^2^, day one to four) and cisplatin (25 mg/m^2^, day two and three) infusions every 21 days until disease progression or adverse effects prohibited further therapy. Pre-therapy ovarian cancer tissues were analyzed by immunohistochemistry for RNR subunit expression as an indicator of cisplatin plus 3-AP treatment response.

**Results:**

3-AP preceding cisplatin exposure in platinum-resistant ovarian cancer cells was not as effective as sequencing cisplatin plus 3-AP together in cell survival assays. Platinum-mediated DNA damage (i.e., γH2AX foci) resolved quickly after cisplatin-alone or 3-AP preceding cisplatin exposure, but persisted after a cisplatin plus 3-AP sequence. On trial, 25 four-day overlapping 3-AP and cisplatin cycles were administered to six women (median 4.2 cycles per patient). 3-AP-related methemoglobinemia (range seven to 10%) occurred in two (33%) of six women, halting trial accrual.

**Conclusions:**

When sequenced cisplatin plus 3-AP, RNR inhibition restored platinum-sensitivity in platinum-resistant ovarian cancers. 3-AP (96 mg/m^2^) infusions produced modest methemoglobinemia, the expected consequence of ribonucleotide reductase inhibitors disrupting collateral proteins containing iron.

**Trial registry:**

ClinicalTrials.gov NCT00081276

## Background

Ovarian cancer is a leading cause of cancer-related mortality in women worldwide, in part due to a greater than 65 percent incidence of intraperitoneal disease persistence, or less than six month disease recurrence after platinum chemotherapy [1]. Chemotherapeutic strategies to overcome ovarian cancer resistance to platinum chemotherapy have included co-administration of paclitaxel or docetaxel, but whether other therapeutics may restore platinum cytotoxicity in “platinum-resistant” cancer remains uncertain [2-4].

Ribonucleotide reductase (RNR) inhibitors such as hydroxyurea, gemcitabine, and 3-aminopyridine-2-carboxyaldehyde-thiosemicarbazone (3-AP) have gained new-found importance as anticancer agents in management of ovarian and cervical cancers [5-9]. RNR catalyzes the rate-limiting step in the *de novo* production of deoxyribonucleoside triphosphates (dNTP) used in DNA synthesis and repair [10]. Functional RNR has two M1 subunits, and either two M2 or two M2b (p53R2) subunits. RNR inhibitors such as hydroxyurea and 3-AP disrupt an essential diferric iron center-generated tyrosyl free radical in RNR M2 or M2b, both prohibiting *de novo* dNTP synthesis and triggering apoptosis [10-12]. When RNR inhibitors are combined with antineoplastic chemotherapy such as cisplatin, enhanced cell death occurs due to a cell’s protracted inability to supply crucial dNTPs at the time of DNA-platinum adduct repair [10]. Much of the controversy in the use of RNR inhibitors with DNA-damaging anticancer therapies centers upon sequencing and timing of the two therapies [8,9].

In this study, we tested whether RNR inhibition by 3-AP preceding cisplatin treatment restores cisplatin cytotoxicity in platinum-resistant ovarian or primary peritoneal cancers by in vitro and ex vivo translational medicine immunohistochemistry assays.

## Materials and methods

### Cell lines, chemicals, and in vitro assays

Two human platinum-resistant ovarian cancer cell lines (SKOV-3, OVCAR-3) were obtained from American Type Culture Collection (Rockville, MD) and cultured at 37°C in a humidified 5% CO_2_ atmosphere. The SKOV-3 and OVCAR-3 ovarian cancer cell lines may be considered refractory to death-provoking effects of platinum agents through expression of multidrug resistance transporters (*mdr-1+*) [13,14]. Eagle’s minimum essential medium (Gibco, Grand Island, NY) supplemented with 10% fetal bovine serum, 1% non-essential amino acids and 1% penicillin/streptomycin was used. Cisplatin and chemicals were purchased from Sigma (St. Louis, MO). 3-AP (NSC #663249) is an investigational RNR inhibitor provided to Case Western Reserve University (Cleveland, OH) under an agreement with the National Cancer Institute Cancer Therapy Evaluation Program (Bethesda, MD) and Nanotherapeutics, Inc. (Alachua, FL). Cells were exposed to therapeutic doses of 3-AP (1, 5, or 10 μM) and cisplatin (5 μM) as indicated, and then, subjected to clonogenic cell survival, RNR activity, and γH2Ax DNA damage assays [10,11]. Because in vivo human minimum inhibitory concentrations of RNR blockade by 3-AP lasts 6 hours [15-18] and cisplatin adduct formation reaches peak six hours after initial cisplatin exposure [19], four conditions were studied: (a) six-hour 3-AP; (b) six-hour cisplatin; (c) six-hour 3-AP followed by six-hour cisplatin sequential exposure (i.e., modeling GOG #126O); or (d) six-hour 3-AP plus cisplatin co-exposure. Drug-containing media was replaced by drug-free media for assays with time points greater than six hours.

Cell viability was assayed by means of colorimetric MTT (3-(4,5-Dimethylthiazol-2-yl)-2,5-diphenyltetrazolium bromide) assay at 24 hours after no treatment or each of the four experimental conditions. Fourteen day clonogenic cell survival also was determined for 300 ovarian cancer cells (per 60-mm dish) after indicated treatments as before [10,11]. Colony fractions (> 50 cells) were normalized by non-treated control plating efficiency. Means (± standard error [SE]) are reported.

RNR activity was measured at six hours and 24 hours after start of cisplatin exposure using a DNA polymerase extension assay quantifying intracellular deoxycytidine triphosphates (dCTP) [10,11]. Means (± SE) are reported. To link RNR activity and relative protein amount, known numbers of ovarian cancer cells were lysed and immunoblotted for RNR M2 (1:1000, rabbit anti-human M2 antibody, Novus Biologicals [Littleton, CO]) and RNR M2b (1:1000, rabbit anti-human p53R2 antibody, Novus Biologicals) protein before (t = 0 h) and 24 hours after start of cisplatin exposure [10]. Ponceau S provided immunoblot loading control [10].

To monitor resolution of cisplatin-mediated DNA damage, manual counts of fluorescent nuclear histone H2AX phosphorylation at Ser-139 (γH2AX), as a surrogate marker of DNA damage, were tabulated using an Olympus BH2-RFCA fluorescence microscope and digital camera (Olympus America Inc., [Melville, NY]) [10,11]. Cells were fixed in 3.7% formaldehyde and stained with mouse anti-human fluorescein isothiocyanate (FITC)-conjugated anti-γH2AX antibody (Millipore, Billerica, MA) used at 1:500 dilution (4 μg/ml) at 24 hours after start of cisplatin exposure. This time point was chosen because 24-hour residual DNA γH2AX foci correlate to cell death probability [20]. Means (± SE) are reported.

### Clinical trial and ex vivo ovarian cancer immunohistochemistry

Gynecologic Oncology Group (GOG) protocol #126O enrolled six women (≥ 18 years of age) with recurrent or persistent epithelial ovarian cancer between July, 2005 and August, 2006. To be eligible for study, women must have had either a treatment-free interval following completion of last cycle of platinum of less than 6 months, or progression during platinum-based therapy. Women received 3-AP as a two-hour continuous intravenous infusion at 96 mg/m^2^ daily for four consecutive days (days 1 to 4), following prior safety/tolerability findings [15-18]. Cisplatin was administered one hour after 3-AP on days two and three as a continuous intravenous infusion at a dose of 25 mg/m^2^ daily (maximum body surface area: 2.0 m^2^). One four-day cycle of 3-AP and cisplatin treatment occurred every 21 days. Adverse toxicity was graded following Common Terminology Criteria for Adverse Events (CTCAE) version 3.0. Due to an expected rise in methemoglobin [15], blood pressure, pulse, respiratory rate and O_2_ saturation by pulse oximeter were monitored prior to and every 30 minutes during 3-AP infusion. Methemoglobin levels were determined for patients with symptomatic dyspnea or hypoxia (≤ 92%) requiring oxygen. The antidote for methemoglobinemia, methylene blue 1 to 2 mg/kg intravenous infusion over five minutes [21], was available during 3-AP infusion. Women were eligible to continue treatment unless there was evidence of unacceptable toxicity or progressive disease. Signed written informed consent was obtained from each woman for treatment. GOG protocol #126O was conducted with approval by local institutional review boards.

Response was evaluated by physical examination and/or imaging every other cycle for the first six months and every six months thereafter. Partial (PR), stable (S), progressive disease (PD), and unconfirmed (U) responses were defined by Response Evaluation Criteria in Solid Tumors (RECIST v1.0). Archived formalin-fixed, paraffin-embedded ovarian cancer samples on charged slides were obtained for all six GOG protocol #126O enrollees. Immunohistochemistry (IHC) was performed using RNR M1 rabbit polyclonal (0.5 mg/mL, 1:200, Abcam, Inc. [Cambridge, MA]), RNR M2 mouse monoclonal (0.5 mg/mL, 1:100, Abcam) and RNR M2b (p53R2) rabbit polyclonal (0.2 mg/mL, 1:250; Abcam) antibodies [7]. Using normal tissue controls to calibrate staining intensity and accepting both cytoplasmic and nuclear staining as positive [7], two pathologists blinded to treatment and response independently scored IHC specimens for M1, M2 and M2b (p53R2) protein positivity: negative 0 (<5%), positive 1+ (5% to <25%), positive 2+ (25% to <75%), and positive 3+ (≥75%). Discrepancies were reviewed and resolved by direct communication.

### Statistical considerations

**Figure 1:**
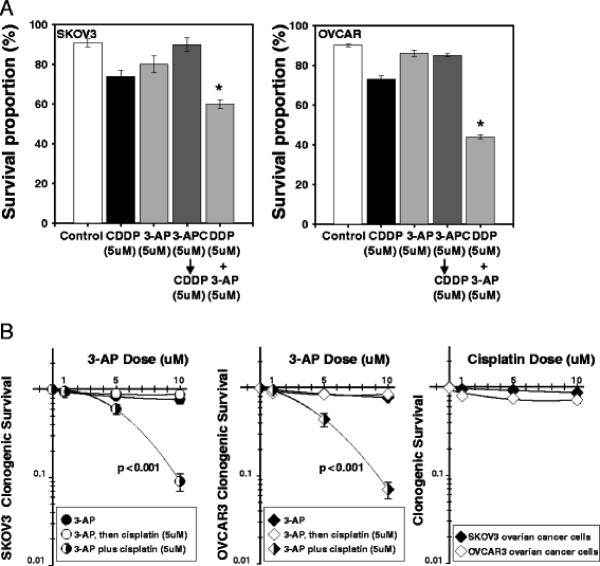
**3-aminopyridine-2-carboxyaldehyde-thiosemicarbazone (3-AP) lowered 6-hour deoxycytidine triphosphates (dCTP) pools and was associated with elevated 24-hour posttherapy M2 and M2b protein.** Panel A: SKOV3 and OVCAR3 cells were treated with cisplatin (5 μM) and/or 3-AP (5 μM) for 6 hours and assayed for dCTP pool by a DNA polymerase extension assay. Cisplatin treatment elevated 6-hour post therapy dCTP levels, indirectly suggesting a rise in RNR activity. dCTP levels 6 hours after cisplatin treatment (and 18 hours after start of 3-AP treatment) were indistinguishable from dCTP levels in cisplatin alone treated cells (*P* = 1.0). When 3-AP was administered alone or co-administered with cisplatin, substantial reduction in dCTP levels were detected 6 hours post therapy (*P* < 0.001, star). Means (± standard error) are reported. Panel B: Immunoblots for M2 and M2b protein and corresponding dCTP level are depicted for control and treated cells (t = 0 h [before] and 24 h [after start of cisplatin]). M2 and M2b showed moderate increase after cisplatin treatment or after ribonucleotide reductase blockade by 3-AP. Corresponding 24-hour dCTP level after cisplatin, an indicator of rise in ribonucleotide reductase activity in response to cisplatin-mediated DNA damage, was higher than baseline (*P* < 0.01, star). Increased ribonucleotide reductase activity occurred in the 3-AP then cisplatin treatment (*P* < 0.01, star). In the three 3-AP treatment groups, 24-hour recovery of ribonucleotide reductase activity after 3-AP inactivation was found. Whether return to baseline or elevated recovery of activity happens because of transcriptional replacement of the M2 and/or M2b subunits or other mechanism is not discerned. Means (± standard error) are reported for RNR activity.

**Figure 2:**
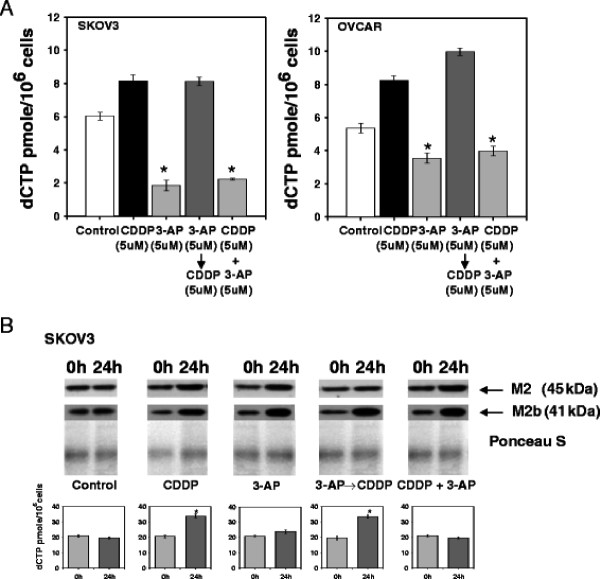
**3-aminopyridine-2-carboxyaldehyde-thiosemicarbazone (3-AP) protracts resolution of γH2AX foci.** Panel A: Manual counts (mean ± standard error) of retained γH2AX foci at 24 hours post therapy (i.e., start of cisplatin exposure) are depicted for SKOV3 and OVCAR3 treated cells. Cisplatin damages DNA resulting in visible γH2AX foci [19]. Cisplatin plus 3-AP treated cells demonstrated an increased number of γH2AX foci, compared to cisplatin alone (*P* < 0.001, star) or 3-AP preceding cisplatin (*P* < 0.001, star) treatment, indicative of retained cisplatin-mediated DNA damage.

One scientific objective was to evaluate the hypothesis that sequencing a RNR inhibitor prior to cisplatin restored platinum-sensitivity in platinum-resistant ovarian cancer cells through a mechanism of 3-AP blocked RNR activity leading to persistent DNA damage. With this objective in mind, in vitro clonogenic survival assays were analyzed by multivariate analysis of variance (MANOVA) statistics for comparison of overall “shapes” of different survival curves across cell treatments [22]. MANOVA statistics (α = 0.05) were computed using statistical software (SPSS 18.0, Chicago, IL). For RNR and γH2AX assays (Figures 1 and 2), ANOVA tests of significance (α = 0.05) were done (SPSS 18.0).

**Figure 3:**
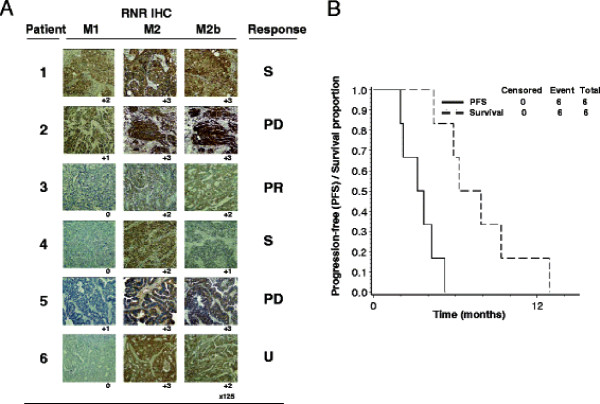
**3-aminopyridine-2-carboxyaldehyde-thiosemicarbazone (3-AP) effectively restored cisplatin sensitivity in “platinum-resistant” SKOV3 and OVCAR3 ovarian cancer cells.** Panel A: Cells were treated with cisplatin (5 μM) and/or 3-AP (5 μM) for 6 hours and assayed by MTT (3-(4,5-Dimethylthiazol-2-yl)-2,5-diphenyltetrazolium bromide) for cell mitochondrial viability at 24 hours after the start of cisplatin exposure. Compared to cisplatin alone, 3-AP alone, and 3-AP preceding cisplatin treatment, a significant cisplatin plus 3-AP interaction was found (*P* < 0.01, star). Panel B: 14-day clonogenic ovarian cancer cell survival was done using cisplatin (5 μM) and a wider therapeutic range of 3-AP (1, 5, or 10 μM). Here too a significant cisplatin plus 3-AP enhancement of cytotoxicity was seen (*P* < 0.001, star), when compared to 3-AP alone or 3-AP preceding cisplatin. Means (± standard error) are reported.

For clinical trial data, descriptive statistics are provided. Monthly decisions regarding the continuance or cessation of trial accrual were guided by a two-stage clinical trial design targeting 23 first-phase and 25 second-phase patients (i.e., anticipated total accrual range 44–51 patients). GOG protocol #126O clinical trial accrual was suspended when two of the first six patients had adverse methemoglobinemia; the trial was not re-opened for accrual. It was decided that an exploratory analysis of collected ovarian cancer samples from the six enrollees was worthwhile to corroborate in vitro cell culture data. To this end, IHC antibody staining scores (0, 1, 2 or 3) for RNR M1, M2, M2b were correlated to treatment response outcome scores (PR = 1, S = 2 and PD [U included] =3) by Spearman rank correlation coefficients determined by a PROC FREQ command (SAS 9.2 [Cary, NC]). It was also concluded that reporting pilot survival data would sharpen investigator thinking about future 3-AP-cisplatin clinical trial design, while at the same time, survival data would not suggest conclusive comment on an overall 3-AP-cisplatin treatment strategy. Thus for the six women treated on trial, estimates of progression-free survival (PFS) and of overall survival (OS) (Figure 3B), defined as time from study entry to first relapse or death, were plotted by the product-limit method of Kaplan and Meier (SAS 9.2).

## Results

### Cell survival assay

**Figure 4:**
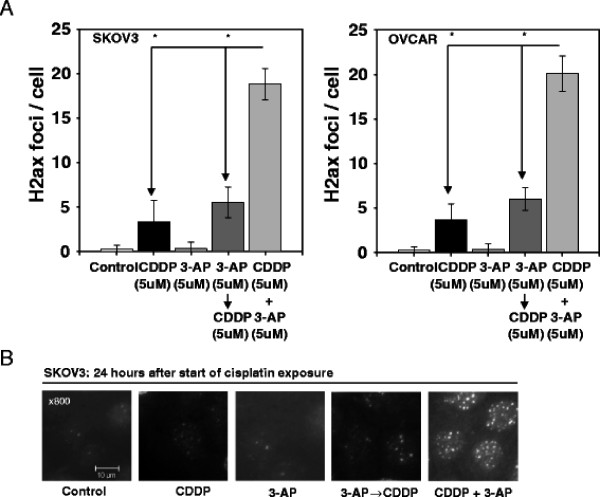
**Pretherapy ovarian cancer ribonucleotide reductase (RNR) M1, M2, and M2b (p53R2) protein is associated with 3-aminopyridine-2-carboxyaldehyde-thiosemicarbazone (3-AP) then cisplatin treatment response.** Panel A: Two pathologists blinded to treatment and outcome scored intensity of ribonucleotide reductase subunit staining. Corresponding best treatment responses are listed (S = stable, PD = progressive disease, PR = partial response, U = undetermined). Panel B: Progression-free survival (PFS) and overall survival are illustrated for six treated patients by 3-AP (96 mg/m^2^) given days 1 through 4 and cisplatin (25 mg/m^2^) given on days 2 and 3 every 21 days. A median 14-week PFS and median 28-week overall survival were observed.

Single and combination agent 24-hour MTT assays of cisplatin and/or 3-AP are presented in Figure 4A. Cytoreduction of 3-AP plus cisplatin was significantly lower than untreated, 3-AP single agent, cisplatin single agent, and 3-AP prior to cisplatin treatment (*P* < 0.01, each). Wider therapeutic range 3-AP alone or added to cisplatin 14-day colony-forming assays are shown in Figure 4B. Sequencing 3-AP starting six hours prior to cisplatin treatment resulted in non-significant cell cytotoxicity compared to 3-AP alone (SKOV-3 *P =* 0.62; OVCAR *P =* 0.70). Co-exposure of cisplatin plus 3-AP significantly reduced platinum-resistant ovarian cancer cell survival (*P <* 0.001, each), suggesting restored platinum sensitivity when RNR is inhibited during an accumulation of cisplatin-mediated DNA damage. In these two cell lines that are *mdr-1*+ and should show relative insensitivity to platinum agents, cisplatin treatment alone resulted in minor cytotoxicity, as shown in Figure 4B.

### RNR activity and immunoblot assays

RNR activity was monitored in Figure 1A by representative intracellular dCTP pools after indicated cisplatin and/or 3-AP treatments. Intracellular dCTP levels rose 6 hours after cisplatin treatment compared to untreated cells (*P* < 0.01, each), suggesting elevated RNR activity after cisplatin-mediated DNA damage. 3-AP treatment alone (*P* < 0.001, compared to untreated cells) or when co-administered with cisplatin (*P* < 0.001, compared to cisplatin-treated cells) lowered six-hour post therapy dCTP levels in both ovarian cancer cell lines. Sequencing 3-AP six hours before cisplatin treatment resulted in elevated dCTP levels, indicative of recovered RNR activity (SKOV-3, *P* < 0.01 *v.* untreated; *P* = 0.98 *v.* cisplatin-treated) or enhanced RNR activity (OVCAR, *P* < 0.001 *v.* untreated; *P* = 0.01 *v.* cisplatin-treated).

Figure 1B depicts relative M2 or M2b protein amount with corresponding RNR activity after cisplatin and/or 3-AP exposure. In cells, a Fe^+2^-3-AP chelate obliterates the tyrosyl free radical in the RNR M2 and RNR M2b small subunits. A testable hypothesis is whether cells perform Fe^+2^ exchange to recover activity quickly without synthesis of new protein and no substantial change in subunit quantity, or rather cells synthesize entirely new ribonucleotide reductase protein measurable as increased protein amount on immunoblot assays. Thus, immunoblot and RNR activity assays were performed on SKOV3 cells removed from the same cell culture plate. A pronounced rise in RNR M2 and RNR M2b subunit quantity and activity is immediately evident from a comparative immunoblot and RNR activity study of cells 24 hours after cisplatin exposure or its untreated counterpart (Figure 1B*P* < 0.01). After 3-AP exposure, rise in RNR M2 and RNR M2b subunit quantity occurred without appreciable change in corresponding RNR activity. In cells conditioned with 3-AP for six hours and then exposed to cisplatin to created DNA damage, RNR activity significantly is higher than untreated cells (Figure 1B*P* < 0.01). From these experiments, the two parameters that determine RNR activity, unperturbed Fe^2+^-stabilized tyrosyl radicals and functional RNR small subunits permissive of proton-coupled electron transfer [23], are renewed by 24 hours. Whether full recovery of ovarian cancer cell RNR activity, anticipated to facilitate cisplatin-mediated DNA damage repair, is attributable to reversible 3-AP pharmacologic inhibition, 3-AP-induced staggered transcriptional replacement either of the RNR M2 or of RNR M2b subunits, or other mechanisms is under investigation.

### γH2AX DNA damage assays

To ascertain whether RNR pharmacologic blockade hampered repair of cisplatin-mediated DNA damage and corresponded to observed cytoreduction (Figure 4), manual counts of γH2AX foci were done among treated cells as shown in Figure 2. Cisplatin-treated ‘platinum-resistant’ ovarian cancer cells had few (but more than untreated cell [*P* < 0.01]) residual 24-hour γH2AX foci. 3-AP treatment alone was associated with no substantial 24-hour residual γH2AX foci (*P* = 1.0). A sequence of 3-AP then cisplatin provided higher γH2AX foci counts than untreated cells (*P* < 0.001) or cisplatin-treated cells (*P* < 0.01). Cells exposed to cisplatin plus 3-AP (*P* < 0.001, each) showed a significantly high number of retained 24-hour γH2AX foci, as compared to any other treatment. While other mechanisms modulating chemoresistance are likely, 3-AP renders cells more vulnerable to DNA damage when co-administered, rather than given prior to, DNA damaging agents. In-depth time course modeling of γH2AX foci formation and resolution kinetics after DNA damaging agent and RNR inhibitor therapy are underway.

### 3-AP then cisplatin in ovarian cancer clinical trial outcomes

Characteristics of six women with epithelial ovarian cancer enrolled on GOG protocol #126O are itemized in Table 1. All patients had received at least one prior chemotherapy regimen; all six women had received prior platinum chemotherapy. The interval from last cycle of platinum to the date of progression on platinum therapy or to cancer recurrence was 0, 22, 89, 122, 139, and 164 days; all were less than the 182 day (6 month) eligibility criterion. A total of 25 cycles (median, 4.2 cycles per patient) of four-day overlapping 3-AP then cisplatin treatment were given. Six patients experienced 18 hematological adverse events and two dose-limiting grade 3 dyspnea adverse events probably attributed to 3-AP infusion were recorded (Table 2).

**Table 1:** Patients characteristics (n = 6)

Characteristic	Number patients (%)
Age (years)	
40-49	1 (16.7)
50-59	4 (66.7)
60-69	1 (16.7)
Race	
African-American	1(16.7)
Caucasian	5 (83.3)
GOG Performance Status	
0	5 (83.3)
1	0 (0.0)
2	1 (16.7)
Ovarian cancer sub-type	
Endometrioid	1 (16.7)
Mixed epithelial carcinoma	1 (16.7)
Serous adenocarcinoma	4 (66.7)
Prior therapy	
Chemotherapy	6 (100)
Median number prior regimens (range)	1(1)
Surgery	6 (100)

GOG = Gynecologic Oncology Group.

**Table 2:** Adverse sequelae after 3-day 3-AP then cisplatin (n = 6)

**Adverse Event Category**	**0**	**1**	**2**	**3**	**4**	**5**
Anemia	1	1	4	0	0	0
Leukopenia	1	2	2	1	0	0
Neutropenia	1	1	2	1	1	0
Thrombocytopenia	3	1	2	0	0	0
Allergy/Immunology	5	0	1	0	0	0
Constitutional	2	3	1	0	0	0
Dermatologic	3	1	2	0	0	0
Nausea	3	1	2	0	0	0
Vomiting	3	0	2	1	0	0
Gastrointestinal	3	2	0	1	0	0
Metabolic	3	1	2	0	0	0
Neurosensory	4	2	0	0	0	0
Other Neurological	4	1	1	0	0	0
Pain	5	0	0	1	0	0
Pulmonary	4	0	0	2	0	0

In one dyspneic patient, on day 1 of the first cycle of 3-AP, blood oxygen saturation fell to 86% and the methemoglobin level rose to 7% (normal methemoglobin levels are 1-2%, [21]). Oxygen supplementation (5 L/min for 14 hours) returned the patient’s blood oxygen saturation to normal and reduced methemoglobin levels to 1.2%. The dose of 3-AP was reduced 25% for the remainder of cycle one. In this patient’s second cycle with the 3-AP dose 25% reduced on day one, blood oxygen saturation again decreased to 80% and methemoglobin rose to 7%. Oxygen supplementation (2 L/min) and methylene blue administration returned the patient’s blood oxygen saturation to 97% and reduced methemoglobin levels to 1.5% within two hours. For days two to four, the 3-AP dose was reduced further by one-third. The patient completed eight more cycles of reduced dose 3-AP therapy without other symptoms or methemoglobinemia. A second patient with dyspnea had a blood oxygen saturation of 90% and an associated 10% methemoglobin level. Oxygen supplementation (2 L/min) resolved the patent’s dyspnea and the methemoglobin level was 1% within two hours. For this patient, no 3-AP dose modification was done; instead, this patient was removed from the study at the recommendation of the treating physician. Among six patients, dose-limiting methemoglobinemia was recorded after two (2%) of 100 3-AP (96 mg/m^2^) intravenous infusions.

Figure 3A depicts pre-therapy ovarian cancer tumor IHC for RNR M1, M2, and M2b (p53R2) proteins. Women achieving partial response (PR), stable disease (S), and progressive disease (PD or U) after overlapping 3-AP then cisplatin treatment were coded 1, 2, and 3, respectively. Spearman rank correlations of response with histological scores were found to be ρ = 0.782 for RNR M2 (*P* = 0.13), ρ = 0.433 for M2b (p53R2) (*P* = 0.50) and ρ = 0.233 for M1 (P = 0.70). A partial response was seen in one (17%) of six women whose disease was deemed platinum-refractory. Moreover, stable disease was recorded in two (33%) of six women whose disease was listed as platinum-refractory. The duration of partial or stable disease response was less than six months, and the longest progression-free interval observed was 20 weeks. PFS (median 14 weeks) and OS (median 28 weeks) are displayed in Figure 3B. All six (100%) women have died. All six women died of progressive ovarian cancer disease.

## Discussion

RNR inhibitors have shown high clinical activity and favorable toxicity profiles when co-administered with cytotoxic anticancer therapies, such as cisplatin and radiation [7,8]. Use of RNR inhibitors to improve cytotoxic anticancer agent response is not new [7,8,24-30]. However, the optimal way to integrate RNR inhibitor therapy into cytotoxic anticancer regimens involving cancer cell DNA damage remains unknown. Several clinical trials have shown lower than anticipated anticancer responses when sequencing RNR inhibitors before cytotoxic therapy [27-29]. Other clinical trials have shown substantial gains in therapeutic efficacy when RNR inhibitors are sequenced after cytotoxic therapy, perhaps most conspicuous when RNR inhibitors are administered after irradiation [7,8,24-26]. Administration of 3-AP after a DNA damaging agent has emerged, over time of its clinical development, as the more clinically relevant cytotoxic sequence [7,10-11].

Here, we interrogated whether sequencing 3-AP prior to cisplatin better restored platinum-sensitivity in platinum-resistant ovarian cancer. Our findings that cisplatin plus 3-AP led to substantial DNA damage (i.e. increased number of γH2AX foci), led to impaired RNR activity when dNTPs were most demanded, and led to significant cytoreduction in platinum-resistant ovarian cancer cells are clinically relevant. This is especially noteworthy considering the modest clinical activity seen among the six women with platinum-resistant ovarian cancer treated by an overlapping four-day 3-AP then cisplatin sequence. Sequencing 3-AP, and therefore targeted inhibition of RNR after cisplatin treatment, not only increases cisplatin-mediated DNA damage in “platinum-resistant” ovarian cancer cells, but also blocks *de novo* dNTP supply when needed most for cisplatin-DNA adduct repair. Such data mimics radiochemotherapy sensitizing properties of 3-AP in cervix cancer cells [10,11]. Our study would be strengthened by a more rigorous molecular interrogation of RNR inactivated by 3-AP, subsequent recovery of RNR activity, and high RNR activity facilitated cisplatin-induced DNA damage repair in “platinum-resistant” cancer cells.

The finding of relatively high levels of RNR M2 in non-responders is of interest. RNR M2 is a short-lived protein as a consequence of sequences promoting proteosome-dependent breakdown in late mitosis [31]. It is reasonable to speculate that “platinum-resistant” ovarian cancers with high RNR M2 levels may have a large S-phase population, escaping cisplatin-mediated cytotoxicity through enhanced repair of stalled forks formed at cisplatin-DNA adducts during S-phase DNA replication [32]. Alternatively, IHC-detected elevated levels of intracellular RNR M2 may reflect elevated RNR activity which would facilitate cisplatin-DNA adduct repair through timely on-demand supply of *de novo* dNTPs [10]. Current research is exploring each intriguing possibility more closely.

Lastly, dose-limiting methemoglobinemia was observed in two women after 3-AP intravenous infusion, halting GOG protocol #126O clinical trial accrual. The mechanism of RNR inhibition by 3-AP is via inactivation of the tyrosyl free radical within the M2 or M2b (p53R2) small subunits [23,33,34]. Basically, this is a molecular interaction of a Fe^2+^-3-AP chelate and of oxygen generating local reactive oxygen species capable of annihilating the nearby tyrosyl free radical. In a similar manner, a Fe^2+^-3-AP chelate interferes with methemoglobin-hemoglobin cycling. Oxygenated Fe^2+^ hemoglobin oxidizes to Fe^3+^ methemoglobin and superoxide at a rate of 3% per day. Methemoglobin is normally reduced to hemoglobin by cytochrome b5 reductase, accounting for 94% of recycling methemoglobin to hemoglobin [35]. Methemoglobin is thus maintained at a level of 1% of total hemoglobin. Symptomatic dyspnea occurs in fit adults when methemoglobin blood levels reach 25%, but symptoms could occur at much lower levels of methemoglobin when co-morbid conditions exist. Dose-limiting methemoglobinemia was encountered twice in six women, but among a total of 100 individual 3-AP infusions. Mechanistically, 3-AP methemoglobinemia is expected to be independent of 3-AP mediated augmentation of DNA damaging agent effects. Gains in best sequenced and timed RNR inhibitor and DNA damaging agent therapy should translate into overall clinical anticancer benefit without undue methemoglobin toxicity. For example, in patients with cervical cancer where 3-AP is administered immediately after irradiation for maximal radiosensitizing effect and on a different day from cisplatin to lessen “off-target” toxicity from a cisplatin-3-AP effect, symptomatic methemoglobinemia is not encountered [7].

## Conclusions

When sequenced cisplatin plus 3-AP, inhibition of ribonucleotide reductase restored platinum-sensitivity in otherwise platinum-resistant ovarian cancers. 3-AP (96 mg/m^2^) infusions produced modest methemoglobinemia. Pre-clinical studies and phase 1 human trials are needed to determine if RNR inhibitor treatment should be initiated together or promptly after platinum treatment to enhance cytoreduction in other “platinum-resistant” cancers.

## Abbreviations

RNR: Ribonucleotide reductase; dNTP: Deoxyribonucleoside triphosphates; GOG: Gynecologic Oncology Group; CTCAE: Common Terminology Criteria for Adverse Events; PR: Partial response; S: Stable; PD: Progressive disease; U: Unconfirmed; RECIST: Response Evaluation Criteria in Solid Tumors; IHC: Immunohistochemistry; PFS: Progression-free survival; OS: Overall survival.

## Competing interests

The authors declare that they have no competing interests.

## Authors’ contributions

CK carried out the MTT and clonogenic assays, the ribonucleotide reductase activity assay, the γH2AX assay, and drafted this manuscript. TR performed statistical analyses for in vitro assays and immunohistochemistry, and participated in the drafting of this manuscript. FAK carried out the immunohistochemistry, the scoring of staining intensity, and participated in the drafting of this manuscript. JF enrolled patients in the clinical trial and assisted in the drafting of this manuscript. OA recruited patients for clinical trial participation and assisted in the drafting of this manuscript. AB recruited patients for the clinical trial and assisted in the drafting of this manuscript. LU designed the clinical trial, performed toxicity assessments, and helped draft this manuscript. All authors read and approved the final manuscript.
